# Antitumor activity of a novel dual functional podophyllotoxin derivative involved PI3K/AKT/mTOR pathway

**DOI:** 10.1371/journal.pone.0215886

**Published:** 2019-09-26

**Authors:** Yongli Li, Tengfei Huang, Yun Fu, Tingting Wang, Tiesuo Zhao, Sheng Guo, Yanjie Sun, Yun Yang, Changzheng Li

**Affiliations:** 1 College of Basic Medical Science, Sanquan College of Xinxiang Medical University, Xinxiang, Henan, P. R. China; 2 College of Basic Medical Science, Xinxiang Medical University, Xinxiang, Henan, P. R. China; 3 Experimental Teaching Center of Biology and Basic Medicine, Sanquan College of Xinxiang Medical University, Xinxiang, Henan, P. R. China; University of Leeds, Faculty of Medicine and Health, UNITED KINGDOM

## Abstract

The progression of cancer through local expansion and metastasis is well recognized, but preventing these characteristic cancer processes is challenging. To this end, a new strategy is required. In this study, we presented a novel dual functional podophyllotoxin derivative, 2-pyridinealdehyde hydrazone dithiocarbamate S-propionate podophyllotoxin ester (Ptox^Pdp^), which inhibited both matrix metalloproteinases and Topoisomerase II. This new podophyllotoxin derivative exhibited significant anti-proliferative, anti-metastatic that correlated with the downregulation of matrix metalloproteinase. In a xenograft animal local expansion model, Ptox^Pdp^ was superior to etoposide in tumor repression. A preliminary mechanistic study revealed that Ptox^Pdp^ induced apoptosis and autophagy via the PI3K/AKT/mTOR pathway. Furthermore, Ptox^Pdp^ could also inhibit epithelial–mesenchymal transition, which was achieved by downregulating both PI3K/AKT/mTOR and NF-κB/Snail pathways. Taken together, our results reveal that Ptox^Pdp^ is a promising antitumor drug candidate.

## Introduction

Tumor progression through local expansion and metastasis has been well documented. An approach to efficiently inhibit tumor metastasis is urgently required in clinical practice. Numerous strategies have been proposed to address this; although improvements have been achieved in cancer treatment, efficient approaches for different cancers are still lacking and need to be solved. Topoisomerases are highly enriched in rapidly proliferating cells, and play a crucial role in replication, transcription, and chromosome segregation [1,2]. Etoposide as a topoisomerase (Topo) II inhibitor is widely used in clinical practice. Etoposide is a podophyllotoxin (Ptox) derivative isolated from the Podophyllum species [3], but severe side-effects and multidrug resistance (MDR) often result in treatment failure. To overcome the current limitations, numerous approaches have been made to modify the structure of Ptox [4], including esterification and amination at position 4 to produce 4′-demethylepipodophyllotoxin (DMEP) [5], C-S bond modified aromatic heterocyclic podophyllum derivatives [6], acyl thiourea derivatives of epipodophyllotoxin [7], pyridine acid ester derivatives of Ptox [8], halogen-containing aniline Ptox derivatives [9], and combination of 5-Fu with DMEP derivatives [10]. These modifications were achieved using a molecular hybridization strategy [11,12], and some of the derivatives displayed much better cytotoxic activity than etoposide at the cellular level.

It is well-known that tumor metastasis is responsible for approximately 90% of all cancer-related deaths [13]. In the early stages, the metastatic tumor cells secrete signaling molecules to recruit myeloid cells, that later differentiate into tumor associated macrophages, providing chemokines and metalloproteinase to promote both tumor cell growth and degrade the extracellular matrix (ECM) [14]. The soluble components, immunocytes, stromal cells, and ECM comprise the tumor microenvironment, which has an important effect on tumor proliferation and metastasis. In the tumor microenvironment, the metalloproteinases, such as matrix metalloproteinases (MMPs) and Lysyl oxidase, are either zinc or copper containing enzymes, function to degrade the ECM and correlate with distal metastasis [15,16]. In addition, cancer cells have a high demand for iron and copper in order to maintain robust cell proliferation and metastasis, and therefore, depletion of metal ions, or partial inactivation of metallozymes using metal chelator, is one option to inhibit the proliferation and metastasis of cancer cells [17].

In view of the above mentioned features of tumors, both tumor cells and the irrespective microenvironment should be considered in drug design. In the present study, we proposed a new strategy that combined the structural unit of a metal ion chelator with a Topo II inhibitor, in an attempt to achieve the goal of both inhibiting tumor cell growth via Topo II inhibition and inactivating metalloproteinase by chelation, when administered intravenously. To this end, dithiocarbamates were chosen, not only for their strong chelating ability toward metal ions and proven ability to inactivate metalloenzymes [18,19], but also because of their prospective use in cancer therapy [20–23]. On the other hand, topoisomerases were chosen since it is well-known that they play important roles in cell proliferation, and that inhibition of topoisomerases, especially topoisomerase II, can significantly suppress tumor growth. It is also well-known that podophyllotoxin (Ptox) is a good structural unit as a topoisomerase II inhibitor. In this study, a novel compound, 2-pyridinealdehyde hydrazone dithiocarbamate S-propionate podophyllotoxin ester (Ptox^Pdp^) was prepared using multi-step reactions based on a hybridization strategy. As expected, the new compound exhibited significant inhibition of both Topo II and MMPs in vitro. A growth inhibition assay against liver cancer cells in vitro revealed that Ptox^Pdp^ displayed a better anti-proliferative effect than the parent compounds, 4′-demethylepipodophyllotoxin and etoposide, and similar results were observed in a xenograft model. Moreover, Ptox^Pdp^ exhibited a significant anti-metastatic effect and inhibition in a wound healing and invasion assay, which likely correlated with MMP inhibition. In view of the fact that Ptox^Pdp^ induced excess ROS within cells, the correlation between ROS and apoptosis, and signaling pathways was further analyzed. The preliminarily data revealed that the antitumor activity of Ptox^Pdp^ was manifest through multiple pathways including alterations in apoptosis, autophagy and the PI3K/Akt/mTOR pathway. Interestingly, Ptox^Pdp^ also could reverse the epithelial-mesenchymal transition (EMT) in liver cancer cells, which was achieved by downregulation of both the NF-κB/Snail and PI3K/AKT/mTOR pathways; this feature is first reported for an etoposide derivative

## Materials and methods

### Materials

MTT, ethidium bromide (EB), 2,2’-di-pyridylketone, RPMI-1640, and other chemicals were purchased from Sigma-Aldrich. Fetal bovine serum was purchased from Every Green Zhejiang Tianhang Technology Co. Ltd. (Hang Zhou, China). Anti-NF-κB antibody was obtained from Proteintech Group (Wuhan, China); Antibodies against vimentin, slug, caspase 3, β-actin, Bax, and Bcl-2 were purchased from Boster (Wuhan, China). Antibodies against AKT, p-AKT, mTOR, E-cadherin, and Gapdh were purchased from Enogene (Nanjing, China). 4′-demethylpodophyllotoxin was purchased from Shanghai PureOne Biotechnology (Shanghai, China)

### Preparation of pyridinealdehyde hydrazone dithiocarbamate S-propionic acid podophyllotoxin ester (Ptox^Pdp^)

Ptox^Pdp^ was synthesized using a four step reaction: the first three-step reaction was similar to a previously reported protocol, except that 2-pyridylaldehyde was used as starting material [24]. Briefly, hydrazine dithiocarbamate (compound I) was synthesized by reacting an equimolar amount of carbon disulfide (1mmol) with hydrazine (1 mmol) in KOH containing ethanol (10 mL) in an ice bath for 1h. Following this, the reaction mixture without further separation was mixed with an equimolar amount of 2-pyridylaldehyde (1 mmol) and the resulting mixture was refluxed for 1h. After cooling, the red-brown solid (compound II) was filtered and washed with cold ethanol. TLC analysis showed one spot. Next, the red-brown (2-pyridylhydrazone dithiocarbamate, 1mmol)) was dissolved in absolute ethanol, and reacted with 3-bromo propionic acid at room temperature for 1h. The yellow solid product (compound III) was filtered and then washed with ethanol. Compound IV (Ptox^Pdp^) was obtained by reacting compound III with 4′-demethylpodophyllotoxin with DCC/DMAP catalysis in absolute CH_2_Cl_2_. TLC traced during the period of reaction. ^1^HNMR (Broker, ppm): NMR: ^1^HNMR (DMSO-d6): 14.95 (s, 1H), 8.81 (d, 1H, J = 4Hz), 8.57 (m, 1H, J = 4Hz), 8.00 (m, 3H, J = 4, 8Hz), 7.51 (m, 3H, J = 4, 8 Hz), 6.96 (s, 1H), 6.54 (s, 1H), 6.31 (s, 2H), 6.01 (s, 2H), 5.48 (d, 1H, J = 8Hz), 4.75 (dd, 1H, J = 4 Hz), 4.60 (dd, 1H, J = 4Hz), 4.36 (d, 1H, J = 8Hz), 4.20 (dd, 1H, J = 8Hz), 3.61 (s, 6H), 3.30(d, 2H, J = 4Hz), 2.82 (m, 1H, J = 4 Hz). ^13^CNMR (100 MHz, DMSO-d6): 174.96, 169.61, 162.31, 151.33, 147.67, 147.60, 147.56,147.37, 146.68, 139.39, 137.40, 135.15, 133.85, 133.72, 130.74, 127.24, 125.15, 120.00, 110.38, 110.20, 109.81, 107.86, 101.40, 67.76, 64.74, 56.38, 43.75, 40.48, 38.69, 36.06, 31.08. ESI-MS (microTOF-Q III, Brucker): molecular composition: C_31_H_29_N_3_O_9_S_2_; m/z: 674.1239 (M+Na, calculated: 674.1243).

### DNA Topo II activity assay

The nuclear extract from HepG2 cell was prepared as previously described [25]. Nuclear extract (0.4 μg) was added to the Topo reaction mixture containing 10 mM Tris-HCl (pH 7.5), 1 mM EDTA, 1 mM ATP, 150 mM NaCl, 0.1% BSA (bovine serum albumin), 5% glycerol, and 0.4 μg pUC18 and 1–3 μL of test agent (1 mM Ptox^Pdp^ in 8% DMSO) in a final volume of 20 μL. Following incubation at 37°C for 30 min., the reaction was terminated by adding 5 μL of stopping buffer (10% SDS, 0.025% bromophenol blue and 10% glycerol). The reaction products were analyzed by electrophoresis using a 1% agarose gel in a TBE buffer (89 mM Tris-HCl, 89 mM boric acid and 62 mM EDTA) containing 0.1% SDS at 45 V for 3 h. The gel was stained with ethidium bromide (0.5 μg/mL) and photographed using a short wavelength UV lamp on a Tocan 360 gel scanner (Shanghai Tiancheng Technology Inc, China). The assay was performed in duplicate.

### Molecular docking

The structure of human type II Top (3QX3) was obtained from RCSB Protein Data Bank. The structure of Ptox^Pdp^ was generated by Chemdraw (Chemdraw Ultra 8.0, Cambridge Soft, USA). Energy minimization was conducted using Chem3D (Ultra 8.0, Cambridge Soft, USA) [26]. PyMol and LigPlot were used to display the conformation and interactions [27,28]

Molecular docking studies were performed using AutoDock Vina and AutoDock Tools based on the recommended procedure [29]. The grid box was set to the center of the etoposide model, and the grid box size for Ptox^Pdp^ was set to 22, 24, and 28 for the X, Y, and Z axes, respectively. Ptox^Pdp^ was set as a flexible ligand by using the default parameters of the AutoDock Tool. The optimal conformation of the ligand was generated using AutoDock Vina.

### Migration assay

The inhibition of tumor cell migration by Ptox^Pdp^ was determined using a wound-healing migration assay [30]. Briefly, HCCLM3 cells were allowed to grow to full confluence in 24-well plates, after which “wounds” were created using a sterile pipette tip. Following this procedure, the cells were rinsed twice with PBS to remove unattached cells. Fresh medium containing 10% fetal bovine serum and various concentrations of Ptox^Pdp^ was then added. The image was photographed and distances between scratches measured under inverted microscopy (×20 magnification).

### Invasion assay

As described previously [31], transwell chambers (Corning) with 8-μm-pore membranes coated with Matrigel were used to perform the invasion assay. Briefly, after overnight pretreatment with Ptox^Pdp^ in a 6-well plate, the HCCLM3 cells were starved for 12 h in serum-free medium. Following this, the cells were collected and re-suspended as a single cell suspension. In total, 3×10^4^ cells in 100 μL of serum-free medium were added to the upper chamber, and 600 μL of complete medium was added to the lower chamber. Following incubation for 18 h at 37°C, the invading cells were fixed in 4% paraformaldehyde and stained with 0.1% crystal violet and photographed under a microscope (or counted manually). The percentage of inhibition was expressed using control wells as 100%.

### Gelatin zymography assay

Gelatin zymography was performed as previously described [32]. Conditioned media were collected from HCCLM3 cells after culture for 24 h in serum free medium with or without Ptox^Pdp^. After collection, the media were centrifuged to pellet any insoluble material. The protein concentration in the conditioned media was quantified using the Bradford method. Equal amounts of conditioned media were mixed with sample buffer and applied to 10% SDS polyacrylamide gel copolymerized with 1 mg/mL of gelatin. After electrophoresis, the gel was incubated in re-naturing buffer (50 mM Tris HCl, pH7.5, 2.5% Triton X-100, 200 mM NaCl, 10 mM CaCl_2_ and 1 μM ZnCl_2_), followed by a 24 h incubation with developing buffer (50 mM Tris-base, 200 mM NaCl, 10 mM CaCl_2_ and 1 μM ZnCl_2_, 0.02% NaN_3_). The gel was then stained with 0.25% Coomassie blue R-250 for 1 hour and destained with 10% methanol with 5% acetic acid. Clear bands against a dark blue background indicated where the protease had digested the gelatin and were taken to be indicative of protease activity.

### Cytotoxicity assay (MTT assay)

A 10 mM Ptox^Pdp^ solution in 80% DMSO was diluted to the required concentration with cell culture medium. The MTT assay was conducted as previously described [25]. Briefly, 5×10^3^/mL HepG2 (or HCCLM3) cells in exponential-phase were seeded at equivalent cell densities into a 96-well plate, and various amounts of Ptox^Pdp^ were added to the adhered cells. After 48 h incubation at 37°C in a humidified atmosphere of 5% CO_2_, 10 μL of MTT solution (5 mg/mL) was added to each well, followed by a further 4 h incubation. The cell culture was removed and 100 μL DMSO was added to each well to dissolve the formazan crystals. The measurement of the solutions absorbance, to give a reading of the number of live cells, was performed on a microplate reader (MK3, Thermo Scientific) at 570 nm. Percent growth inhibition was defined as the percent absorbance inhibition within appropriate absorbance in each cell line. The same assay was performed in triplicate.

### Cell clonogenic assay

To mimic individual cell development into macroscopic cell clones, a cell clonogenic assay was performed as previously described [33]. HepG2 cells were seeded into a 6-well plate and allowed to grow for 24 h. Cells were then treated with different concentrations of Ptox^Pdp^ for 24 h, after replaced the media, and allowed the cells grow in normal media for 14 d. Next the colonies were fixed in 3.7% paraformaldehyde, stained with 0.1% crystal violet and counted manually.

### HepG2 xenograft animal model

A xenograft model was established in BALB/c female nude mice, 4–6 weeks of age and weighing 19–21 g (Beijing Vital River Laboratory Animal Technology Co., Ltd, China). The mice were kept in specific-pathogen-free environments at 26–28°C under 40–60% relative humidity, with a 10-h light/14-h dark cycle, and food and water were provided ad libitum. HepG2 cells (1 × 10^7^ in 0.1 mL PBS) were inoculated into the mammary fat pads of Balb/c nude mice. When tumors were palpable (on day 5, 60 ~ 188 mm^3^), the 12 male mice were divided randomly into four groups (n = 3 per group), Thereafter, Ptox^Pdp^ (normal saline plus DMSO and etoposide) at 0.5 mg/kg, 1 mg/kg was administered via intralesional injection every six days. Tumor growth was measured every two days with a caliper and the volume was calculated according to the formula: V = 1/2 (d_length_ × d^2^_width_) [34]. On day 20, mice were killed via cervical dislocation under deep isoflurane anesthesia (3% for induction, until breathing of the animal was slow) [35]. Each excised tumor was weighed individually and recorded using a digital camera. Half of each primary tumor was snap frozen in liquid nitrogen and stored at -80°C. The other half was fixed and prepared for immunohistochemical analysis. The animal studies were approved by the Animal Welfare and Ethics Group of the Laboratory Animal Science Department, Xinxiang Medical University.

### Flow cytometry analysis of apoptosis and cellular ROS

Cellular ROS determination was performed based on previously described [36]. Apoptosis was measured using the Apoptosis Detection Kit (Dojindo Laboratories) as company recommended. Briefly, HepG2 cells were treated with Ptox^Pdp^ for 24 h. Following this, cells were collected, washed, and stained with annexin V-FITC and propidium iodide (PI) following the manufacturer’s instruction (Dojindo Laboratories, Japan). The intracellular ROS assay was similar to the above mentioned protocol, except that H_2_DCF-DA was used to stain the cells.

### Comet tail assay

The comet assay tail assay was adapted from the literature, as described [25]. Briefly, HepG2 cells were treated with or without Ptox^Pdp^ (0.75 and 1.5 μM) for 24 h. The cells were collected and embedded in 0.5% low melting-point agarose at a final concentration of 10^4^ cells/mL. The cell suspension (20 μL) was then spread onto duplicate frosted slides that had previously been covered with 1% normal melting-point agarose as a basal layer. Slides were allowed to solidify for 10 min at 4°C, then placed in lysis buffer for 1 h (2.5 M NaCl, 0.1 M ethylene diamine tetraacetic acid (EDTA), 0.01 M Tris-HCl,1% Triton X-100, 10% DMSO, pH 10.0). After lysis, the slides were transferred to an alkaline buffer for 40 min (0.001 MEDTA, 0.3 M NaOH, pH >13.0) to allow the DNA to unwind before electrophoresis at 0.66 V/cm and 300 mA for 30 min. All these steps were conducted in the dark. After neutralization in 0.4 M Tris-HCl, pH 7.4, the slides were stained with EB (20 μg/mL) and covered with a cover-slip. Images were then captured using a fluorescent microscopy.

### Autophagy and lysosomal membrane permeability affected by Ptox^Pdp^

Cells were seeded into a 24-well plate and treated as described above for the cell viability assay. The cells were treated with different concentrations of Ptox^Pdp^ for 24 h. In order to detect the acidic cellular compartment, acridine orange (or LysoTracker Red; Invitrogen) was used, which emits bright red fluorescence in acidic vesicles, but green fluorescence in the cytoplasm and nucleus. After treatment of the cells with Ptox^Pdp^, acridine orange was then added at a final concentration of 1 μg/mL (the recommended concentration of LysoTracker Red) for a period of 15 min. Following PBS washing, fluorescent micrographs were captured using an inverted fluorescence microscope.

### Western blotting analysis

Briefly, 1 × 10^7^ HepG2 cells, treated with or without Ptox^Pdp^, were scraped in lysis buffer (50 mM Tris-HCl, pH 8.0, 150 mM NaCl, 1.0% NP-40, 10% glycerol, and protease inhibitors) and subjected to sonication, followed by centrifugation at 14,000 × g. The clear supernatant was stored at -80°C. Protein concentration was determined using a colorimetric Bio-Rad DC protein assay using the MK3 microplate reader at 570 nm. Proteins (30 μg) were separated on a 13% sodium dodecyl sulfate-polyacrylamide gel at 200 V for 1 h. The separated proteins were subsequently transferred onto a PVDF membrane at 60 V for 1 h. The membrane was washed three times with Tris-buffered saline (TBS) and then blocked for 2 h in TBS containing 0.1% Tween-20 and 5% non-fat skimmed milk. The membrane was incubated at 4°C overnight with the appropriate primary antibody used at a dilution of 1:300 in TBS plus 0.1% Tween-20 (TBST). The membrane was then washed several times with TBST and subsequently incubated with the appropriate HRP-conjugated secondary antibody (1:2,000 in TBST) for 1 h at room temperature. Following washing with TBST, the protein bands were detected using a super sensitive ECL solution (Boster Biological Technology Co. Ltd. China), and visualized using an SYNGENE G:BOX Chemi XX9 (SYNGENE, UK).

### Statistical analysis

Results are presented as the mean ± SEM. Comparisons between two groups were carried out using the two-tailed Student’s t-test. Comparisons between multiple groups were performed by one way ANOVA with Dunnett post-hoc correction. All statistical tests were conducted by using IBM SPSS Statistics (version 19 software). P < 0.05 was accepted as significant.

## Results

### Preparation of 2-pyridinealdehyde hydrazone dithiocarbamate S-propionate podophyllotoxin ester (Ptox^Pdp^)

**Fig 1:**
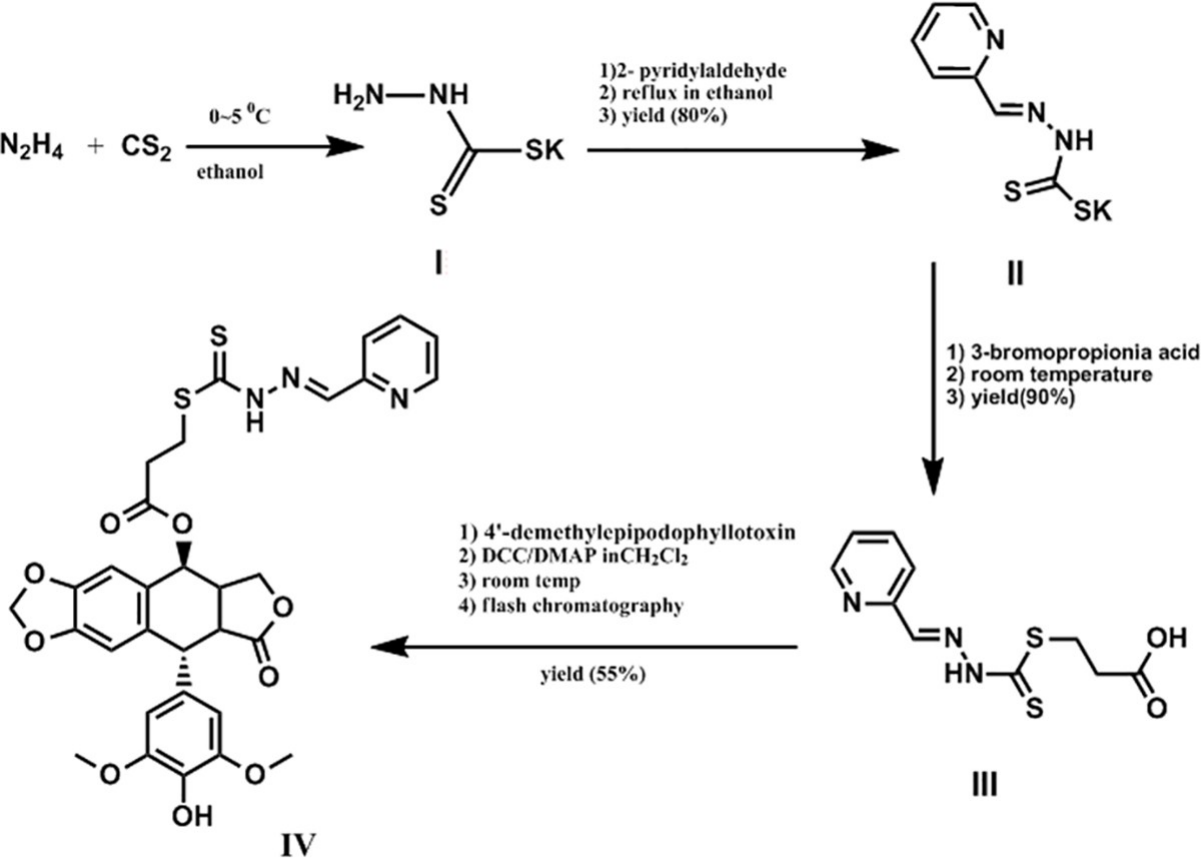
Synthetic route used for the preparation of pyridinealdehyde hydrazone dithiocarbamate S-propionic acid podophyllotoxin ester; the individual reaction conditions are shown in the figure.

Ptox^Pdp^ was prepared using a four-step reaction (**Fig 1**), as described in detail in the Materials and Methods section. The first three-step reaction for the preparation of 2-pyridinealdehyde hydrazone dithiocarbamate S-propionate was followed as previously described [24]. The final product, Ptox^Pdp^, was synthesized by mixing 4′-demethylepipodophyllotoxinwith 2-pyridinealdehyde hydrazone dithiocarbamate S-propionate through catalysis with DMAP/DCC in dichloromethane at room temperature. The reaction process was monitored by TLC. Upon completion, the crude product was subjected to flash chromatography and the isolated product had sufficient purity (>98%, **S1 Fig**). Following NMR and HRMS characterization (**S2–S4 Figs**), Ptox^Pdp^ was identified as the expected product.

### Topo II inhibition of Ptox^Pdp^ and simulation of binding

**Fig 2:**
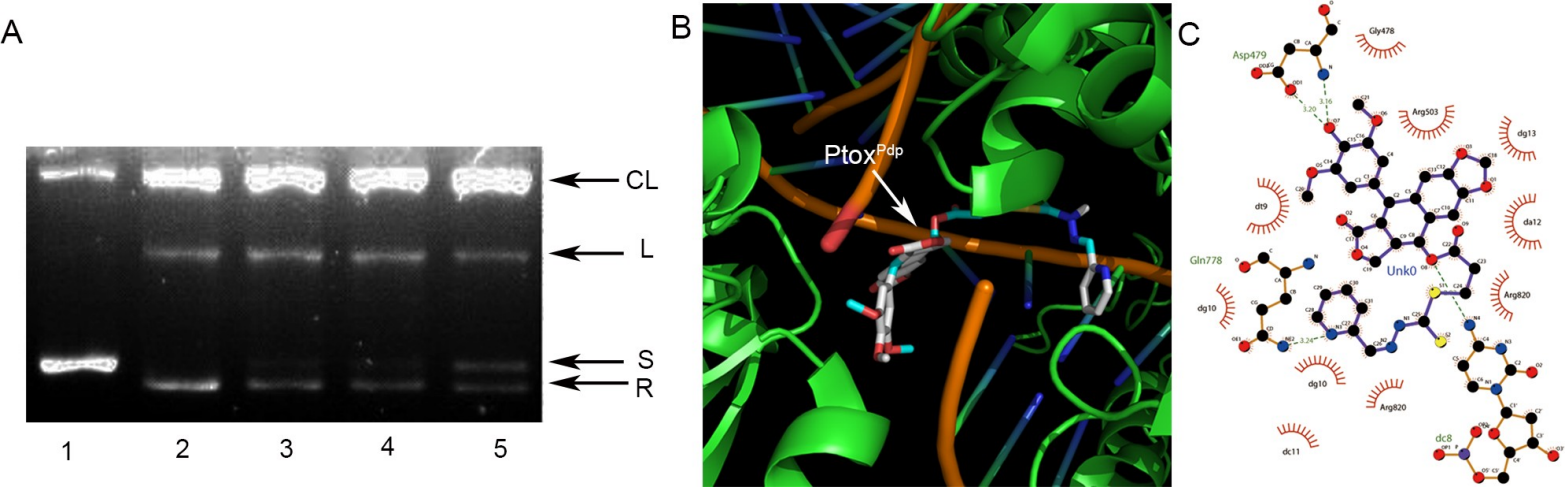
Topo II inhibition and interaction with Ptox^Pdp^. (A) Topo II inhibition by Ptox^Pdp^. Line 1, pUC18; line 2, pUC18 plus nuclear extract; line 3, pUC18 plus nuclear extract and etoposide (50 μM); line 4, pUC18 plus nuclear extract and 0.75 μM Ptox^Pdp^; line 5, pUC18 plus nuclear extract and 1.5 μM Ptox^Pdp^. (B) Ptox^Pdp^ docked to the Topo II-DNA complex. (C) Interaction between Ptox^Pdp^ and nearby residues. CL = cleaved; L = linear; S = supercoiled; R = relaxed DNA. Unk0 = Ptox^Pdp^. Dark green dash lines indicate hydrogen bonds. The eye-like curved dark red lines represent the hydrophobic residues in contact.

Etoposide is a well-known Topo II inhibitor. In order to understand whether the conjugate, Ptox^Pdp^, retained the same activity as etoposide, we first assessed its inhibitory effect on DNA relaxation. Following a previously reported protocol [25], pUC18 plasmid DNA was incubated with a nuclear extract in the absence or presence of various concentrations of Ptox^Pdp^, and the reaction products were subjected to agarose gel electrophoresis. As shown in **Fig 2A**, Ptox^Pdp^ in the presence of ATP also displayed a certain degree of Topo inhibition because the amount of relaxed DNA was decreased compared to the control (**Fig 2A**). Compared to etoposide, Ptox^Pdp^ appeared to be a more potent Topo II inhibitor since higher concentration of etoposide (50 μM) was required to achieve the same level of inhibition observed with Ptox^Pdp^ (or about 66 folds decrease for Ptox^Pdp^ to achieve similar inhibition of etoposide). We next determined whether the binding of Ptox^Pdp^ to Topo II was similar to that of etoposide. To do this, a theoretical simulation was conducted using a molecular docking approach. The human type II Topo crystal structure (PDB ID: 3QX3) was obtained from the RCSB Protein Data Bank. To ensure the accuracy of our docking protocol, etoposide was re-docked into the Topo-DNA complex based on the recommended procedure (**S5 Fig**), next the Ptox^Pdp^ was docked into the Topo II complex (**Fig 2B**); the simulating affinity energy was -13.6 kcal/mol (compared to that of docked etoposide (-14.8 kcal/mol), a slightly weaker interaction was therefore observed), revealing that replacement of the sugar at the 4-position with the dithiocarbamate derivative did not lead to significant changes in affinity or in the nearby molecular environment of the Topo-bound molecule. The interaction of Ptox^Pdp^ with its nearby residues is shown in **Fig 2C**, clearly showing that the natures of the interactions were mainly Van der Waals forces and hydrogen bonds.

### Ptox^Pdp^ inhibits liver cancer cell proliferation

**Fig 3:**
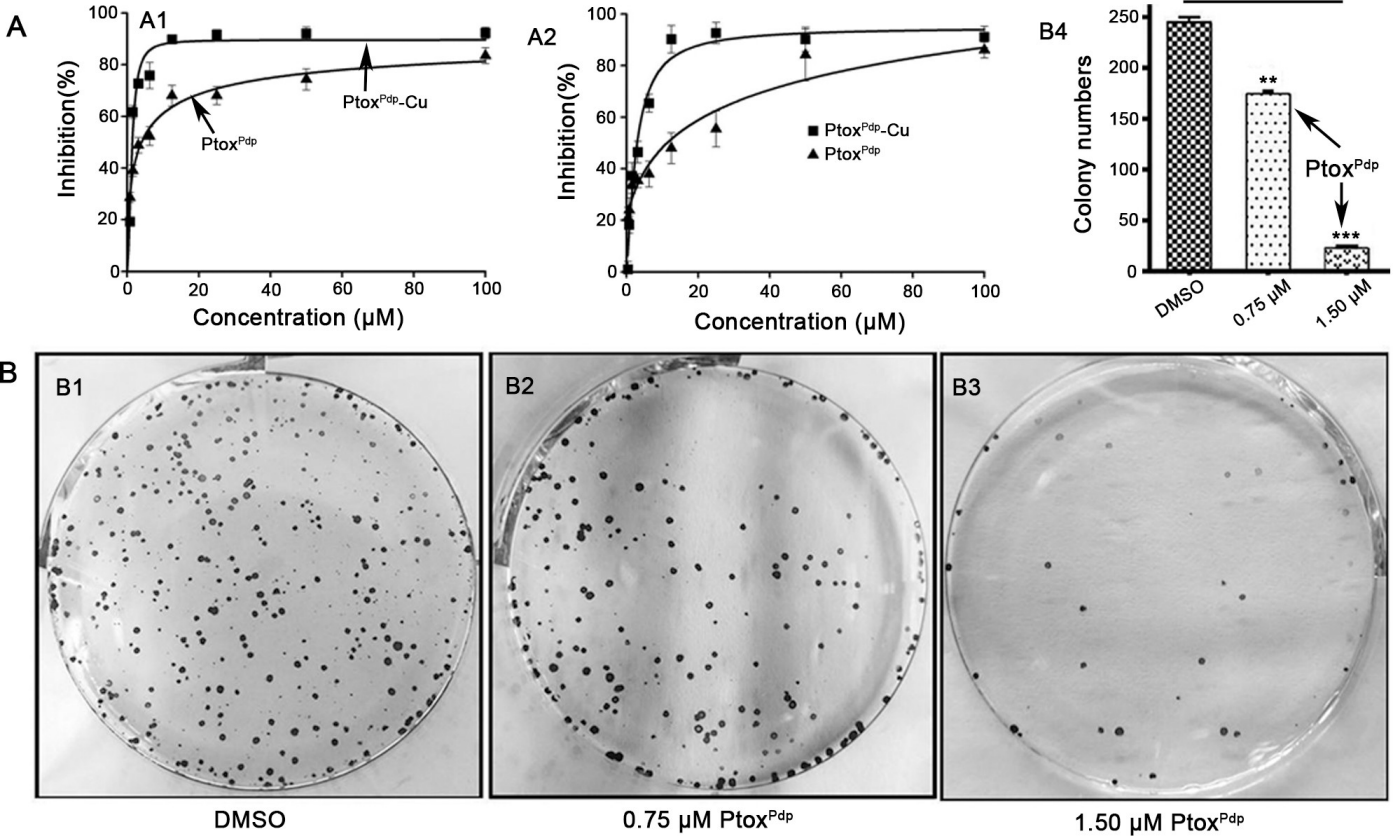
Inhibition of growth and colony formation by Ptox^Pdp^. (A) Growth inhibition by Ptox^Pdp^ of the different hepatocellular carcinoma cells lines. The IC_50s_ for Ptox^Pdp^ and Ptox^Pdp^-Cu were 3.89 ± 0.51 and 1.52 ± 0.20 μM against HepG2 cells, respectively; and 11.01 ± 0.63 and 2.95 ± 0.40 μM against HCCLM3 cells, respectively. (B) Inhibition of HepG2 cell colony formation by the different indicated concentrations of Ptox^Pdp^; the static data are shown in columns (**P < 0.05; ***P < 0.001).

In view of excellent Topo II inhibitory activity of Ptox^Pdp^, growth inhibition assay was required to determine whether cytotoxicity was affected by the structural modification. In addition, liver cancer ranks the fifth in malignant tumor relative death, therefore, the effect of Ptox^Pdp^ on the proliferation of two liver cancer cell lines was preliminarily investigated. The dose-response curves for the two cell lines are shown in **Fig 3A**. Ptox^Pdp^ displayed significant inhibition of growth in the two examined cell lines following statistical analysis (p<0.01, at 0.78 μM). However, a differential effect on the cell lines was also obvious; growth inhibition was achieved at a lower concentration for HepG2 cells (IC_50_ = 3.89 ± 0.51 μM for HepG2 cells, **Fig 3A1**), whereas a much higher concentration was required for HCCLM3 cells (IC_50_ = 11.01 ± 0.63 μM for LCCM3, respectively, **Fig 3A2**), indicating that HepG2 cell was more sensitive to Ptox^Pdp^. Interestingly, Ptox^Pdp^ exhibited better activity than etoposide in inhibiting the proliferation of these liver cancer cells (**S6 Fig**). In view of the chelating feature of Ptox^Pdp^, the inhibition of proliferation by Ptox^Pdp^ in the presence of copper ions was also investigated. Unexpectedly inhibition of growth was dramatically increased in the two cell lines, compared to that induced by Ptox^Pdp^ alone (IC_50_ = 1.52± 0.20 μM for HepG2 and 2.95 ± 0.40 μM for LCCM3, respectively, **Fig 3A**), which stemmed from the formation of Ptox^Pdp^-Cu complexes (**S6 Fig**). The value of IC_50_ determined the concentration used in subsequent experiments. The dose at half of IC_50_, or decreasing exposure time to the cells was adopted in order to decrease cytotoxicity of the agent (see migration and invasion assay). Furthermore the ability of HepG2 cells to form colonies, with or without Ptox^Pdp^ treatment, was examined for a period of two weeks. As expected, Ptox^Pdp^ significantly inhibited colony formation, with ~30% inhibition at 0.75 μM (**Fig 3B2**), while the survival rate was reduced to ~10% at 1.50 μM treatment (**Fig 3B3**). The quantitative analysis in alteration of the colony numbers/view in each group was showed in **Fig 3B4**, clearly there was significant difference between the groups in statistics (p<0.05 for 0.75 μM Ptox^Pdp^, and p< 0.001 for 1.50 μM Ptox^Pdp^).

### Ptox^Pdp^ inhibits cell migration and invasion

**Fig 4:**
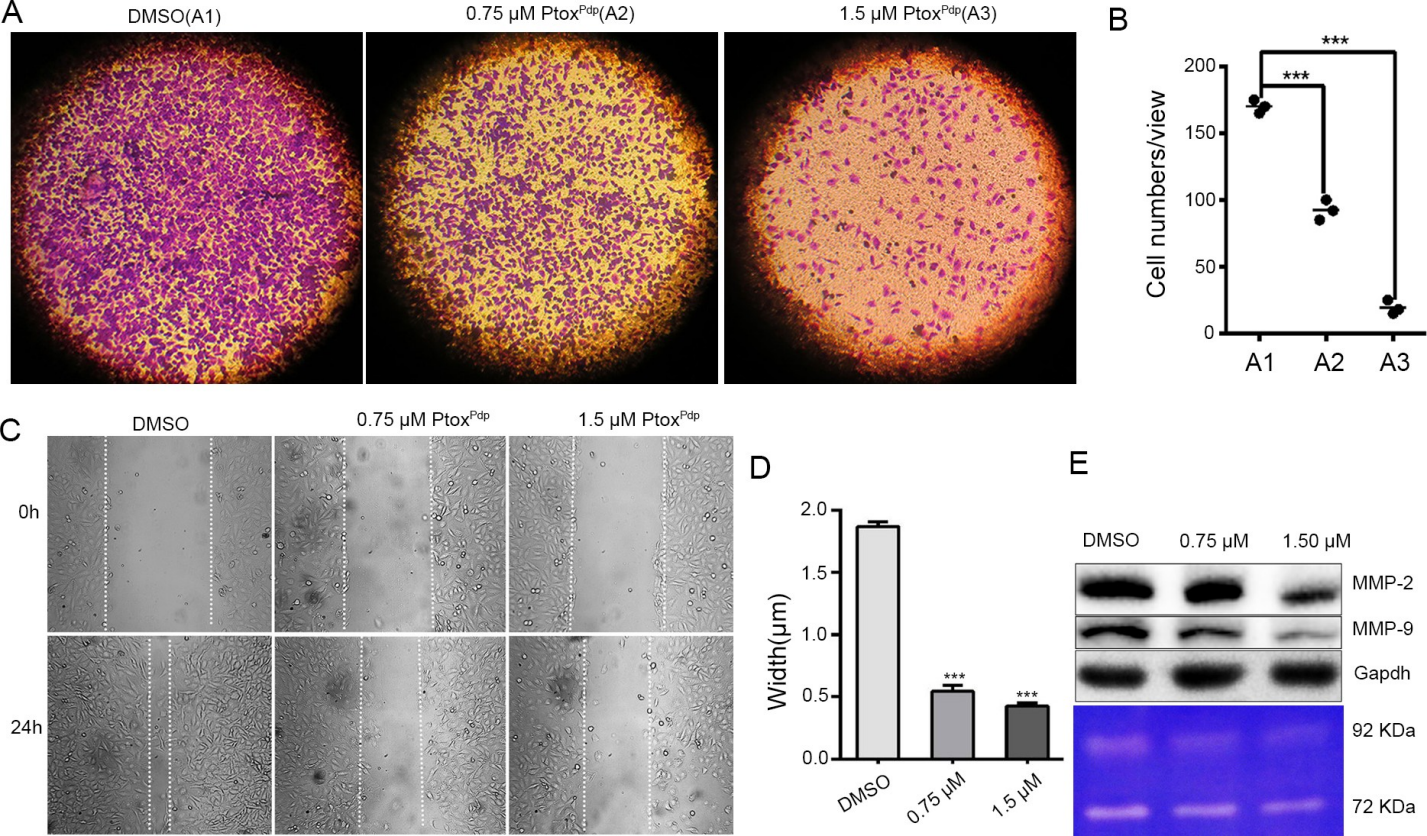
Inhibition of liver cancer cell migration and invasion by Ptox^Pdp^. (A) Inhibition of HCCLM3 cells migration by Ptox^Pdp^. (B) Inhibition of HCCLM3 cell invasion by Ptox^Pdp^ and quantification. The invasive cells were stained with crystal violet. The results were expressed as invasive cell numbers per field of view (mean± 5 SD, n = 6). ***P < 0.001 compared with DMSO-treated group. (C) Wounded HCCLM3 cells were treated with 0, 0.75, or 1.50 μM Ptox^Pdp^ for 18 h. (D) Quantitative statistics analysis of the width of gaps in the wound assay in (C). ***P < 0.001 compared with DMSO-treated group. (E) Western blotting (top) and gelatin zymography (bottom) analyses of matrix metalloprotease inhibition, under the indicated conditions. The object size: 10×10 in **Fig 4A** and 20×10 in **Fig 4C**.

Both cell invasion and migration are of fundamental importance in tumor metastasis and angiogenesis [37]. To determine the effects of Ptox^Pdp^ on the cell invasion, the highly invasion malignant HCCLM3 cell was chosen, and a transwell assay was performed. To minimize the effect of cytotoxicity of Ptox^Pdp^ on invasion and migration, the dose was used at 1/14, 1/7 IC_50_ and shorter exposure time (18 h). As shown in **Fig 4A**, HCCLM3 cells displayed a high invasion capability (**Fig 4A1**). In contrast, Ptox^Pdp^ significantly attenuated the invasion capacity of these cells in a dose-dependent manner (**Fig 4A2 and 4A3**); a quantitative analysis is presented in **Fig 4B**, clearly the numbers of invasive cells into matrix gel were significantly decreased upon exposure of Ptox^Pdp^ to the cells compared to that of DMSO (p < 0.001). A wound-healing model is often widely used to estimate the migration potential of endothelial cells. Therefore, the effect of Ptox^Pdp^ on the migration of HCCLM3 cells was determined. As shown in **Fig 4C**, the migration of HCCLM3 cell across the wound space was inhibited by Ptox^Pdp^ in a dose-dependent manner, or the healing capacity of the cells was markedly inhibited (in terms in width, p < 0.001) compared DMSO with Ptox^Pdp^ (**Fig 4D**), indicating that Ptox^Pdp^ owned certain inhibitory effect on migration and invasion. To understand the potential correlation with MMP inhibition, western blotting and gelatin zymography analysis were conducted. As shown in **Fig 4E**, treatment with Ptox^Pdp^ significantly reduced both MMP-2 and MMP-9 expression and activity, indicating that Ptox^Pdp^ displayed significant inhibition of migration and invasion that correlated with MMP repression.

### Ptox^Pdp^ inhibits HepG2 cell growth in a xenograft cell model

**Fig 5:**
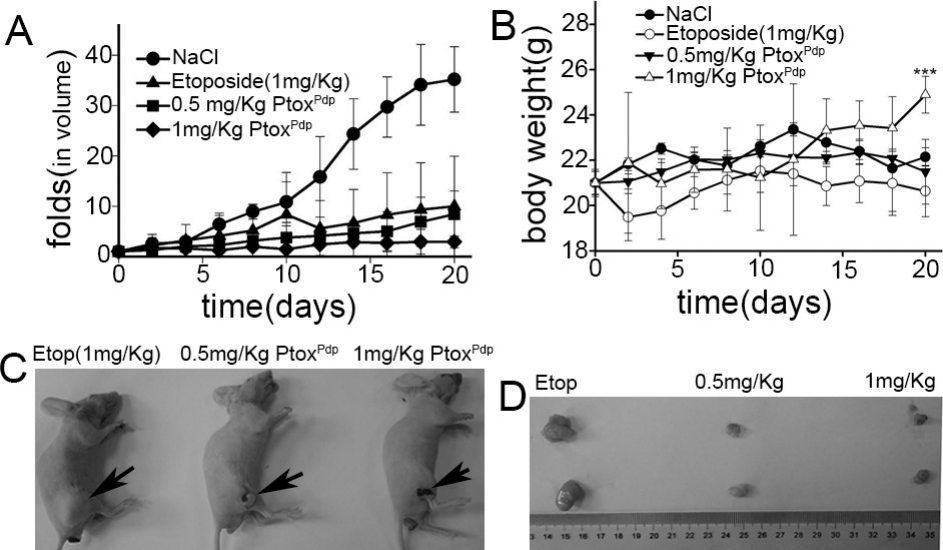
The effect of Ptox^Pdp^ in a HepG2 xenograft animal model. (A) folds increased in tumor volume were defined as the ratio of means of tumor volume to the initial means of tumor volume in the same group; (B) alteration in means of body weight; there was no significant difference before and after treatment within the groups (p = 0.110~0.735), however significance in change of body weight at the end of the study was observed between NaCl and 1mg/Kg treatment group; (C) comparison of etoposide treated with Ptox^Pdp^ in Balb/c nude mice; (D) the excised tumors from different nude mouse in same group, and the group as indicated in the figure. The standard deviation in **Fig 5A and 5B** was obtained by Excell treatment. ***P < 0.05.

Given that Ptox^Pdp^ had such significant biological activity, it was worth evaluating its effects in an animal model. To evaluate the effect of Ptox^Pdp^ on tumor growth *in vivo*, we first established an implantation model by injecting HepG2 cells (5×10^6^) into the flanks of athymic nude mice, and allowed them to develop into a xenograft over a period of two weeks. Once the tumor was palpable, the mice were divided randomly into four experimental treatment groups. Different doses of Ptox^Pdp^ (0.5 and 1 mg/kg) were administered intraperitoneally weekly to two groups, one group received etoposide as the positive control, and the remaining group received normal saline plus DMSO as the negative control. The changes in the average tumor volume for each group are plotted in **Fig 5A**. Clearly, following treatment with Ptox^Pdp^ and etoposide, tumor sizes were dramatically decreased compared to animals dosed with normal saline/DMSO. Interestingly, Ptox^Pdp^ was superior to etoposide in inhibiting tumor growth (**Fig 5A**). Since body weight loss is brought about by cachexia, less bodyweight loss is evidence for reduced cachexia [38]. As shown in **Fig 5B**, Ptox^Pdp^ did not lead to body weight loss compared to controls (p < 0.05, 5 measurements for NaCl; 1 measurement for Ptox^Pdp^), and was better in this regard than etoposide. **Fig 5C** showed that Ptox^Pdp^ inhibited growth of the tumor xenografts. The excised tumor weights from the animals were also recorded, as shown in **Fig 5D**; clearly Ptox^Pdp^ reduced tumor weight more effectively than etoposide. The excised tumors weighed 0.700± 0.21 g for the normal saline/DMSO group, 0.61 ± 0.36 g for the etoposide group, whereas those animals treated with Ptox^Pdp^ weighed 0.116 ± 0.047g (0.5 mg/kg) and 0.043 ± 0.041g, (1 mg/kg) respectively, statistical analysis in tumor weight revealed that the decrease in tumor weight was significant compared Ptox^Pdp^ group with both saline/DMSO group and etoposide group (p<0.05), indicating that Ptox^Pdp^ was a potent antitumor drug against liver cancer.

### Ptox^Pdp^ induces apoptosis

**Fig 6:**
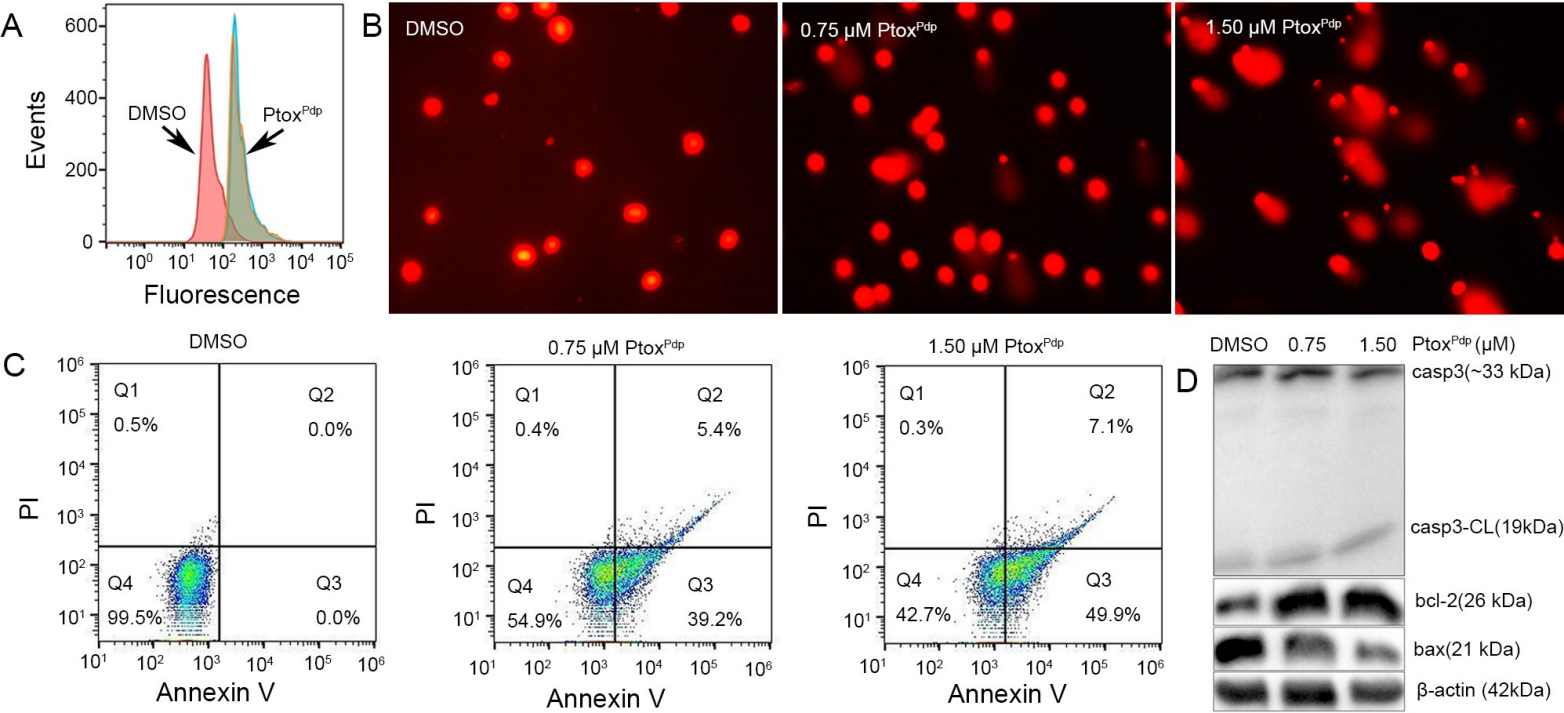
Ptox^Pdp^ induces ROS, DNA fragmentation, and apoptosis in HepG2 cells. (A) Ptox^Pdp^ induces intracellular increases in ROS levels. (B) Ptox^Pdp^ induces DNA fragmentation at the indicated concentrations. (C) Ptox^Pdp^ induces apoptosis, the data were treated by FlowJo software. (D) Western blotting analysis of apoptosis related proteins. The composite images were generated by using Adobe Photoshop. CL = cleaved.

Generation of reactive oxygen species (ROS) is involved in the mechanism of action of many drugs. To understand whether Ptox^Pdp^ has a similar mechanism of action, change in intracellular ROS in cells treated with Ptox^Pdp^ was investigated using flow cytometry. As shown in **Fig 6A**, Ptox^Pdp^ indeed induced ROS generation following exposure to cells for 24 h. The effect of Ptox^Pdp^ on cellular ROS production after different exposure times was also investigated, interestingly ROS generation was similar at all the time points examined (**S7 Fig**), indicating that cytotoxic or anti-proliferative effects of Ptox^Pdp^ correlated with a persistent ROS insult. It has been demonstrated that the ROS cause oxidative damage to DNA and proteins. To explore the correlation between ROS and DNA fragmentation, a comet tail assay was used to assess DNA damage by Ptox^Pdp^. As shown in **Fig 6B**, Ptox^Pdp^, in a concentration-dependent manner, caused an increase in the amount of comet tail compared to controls, indicating that it increased DNA damage. ROS generation and DNA fragmentation lead to cell death, either via apoptosis or necrosis, and an increased presence of phosphatidylserine in the outer leaflet of the plasma membrane is a surface change common to many apoptotic cells [39]. Annexin V which has a high affinity for phosphatidylserine can therefore be used to detect phosphatidylserine in the outer leaflet of the plasma membrane. Accordingly, the Annexin V-FITC/propidium iodide (PI) double-staining technique was employed to determine the effect of Ptox^Pdp^ on apoptosis. As shown in **Fig 6C**, when HepG2 cells were exposed to Ptox^Pdp^ at the indicated concentrations, early apoptosis was increased by 39~49%, and late apoptosis was increased by 5~7%. It was obvious that Ptox^Pdp^ induced a large proportion of HepG2 cell in early apoptosis (p<0.001). To further determine the effect of Ptox^Pdp^ on apoptosis, the levels of apoptosis-related proteins were assessed by western blotting. As shown in **Fig 6D**, Ptox^Pdp^ increased the expression of cleaved caspase-3 and bax, while the expression of bcl-2 was decreased, indicating that the antiproliferative action of Ptox^Pdp^ involved the apoptosis pathway.

### Changes in lysosome (autophagosome) membrane permeability (LMP) and autophagy in cells following exposure to Ptox^Pdp^

**Fig 7:**
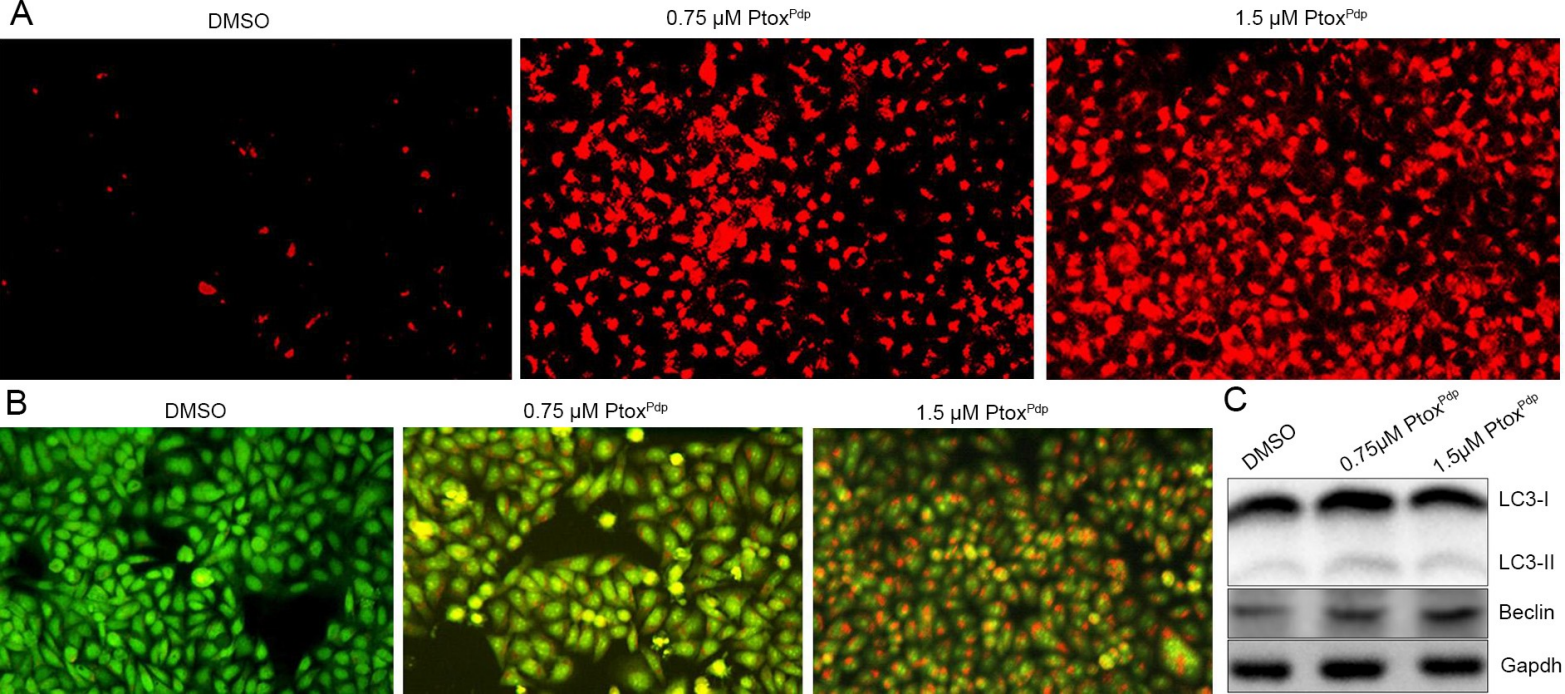
Ptox^Pdp^ induces lysosomal membrane permeability and autophagy in HepG2 cells. (A) Lysosomal membrane permeability assessed using LysoTracker Red staining. (B) Ptox^Pdp^ induces autophagy. The acidic vacuoles in the autophagosomes were stained using acridine orange. (C) Western blotting analysis of changes in the expression of autophagy-related proteins.

Bax translocation to lysosomal membranes can affect lysosomal membrane integrity [40]. In view of the increased bax levels seen in the apoptotic process, it is possible that lysosomal membrane integrity might be affected by bax. To test this hypothesis, Lysotracker Red which can accumulate within lysosomes, was employed to assess lysosomal membrane permeability (LMP) [40]. As shown in **Fig 7A**, the red fluorescence intensities in HepG2 cells were significantly increased in Ptox^Pdp^ treated cells compared to control cells, indicating that Ptox^Pdp^ affected LMP. Furthermore, changes in LMP may be also a response to autophagy, thus the formation of autophagesome was measured using acridine orange staining. As shown in **Fig 7B**, red granular fluorescence in the acidic vacuoles was observed in the Ptox^Pdp^ treated cells, and this increased in a concentration dependent manner, implying that Ptox^Pdp^ induces autophagy. To further examine the involvement of autophagy, the levels of LC3 (microtuble-associated protein light chain 3), an autophagosome marker, were measured by western blotting. As expected, there was an increase in the amount of cleaved LC3-II, and a decrease in uncleaved LC3-I, compared to control cells, indicating that autophagy is involved in the cellular events following exposure to Ptox^Pdp^ (**Fig 7C**).

### The antitumor mechanism of Ptox^Pdp^ involves downregulation of the Akt/mTOR and NF-κB/Snail pathways, accordingly inhibition of EMT

**Fig 8:**
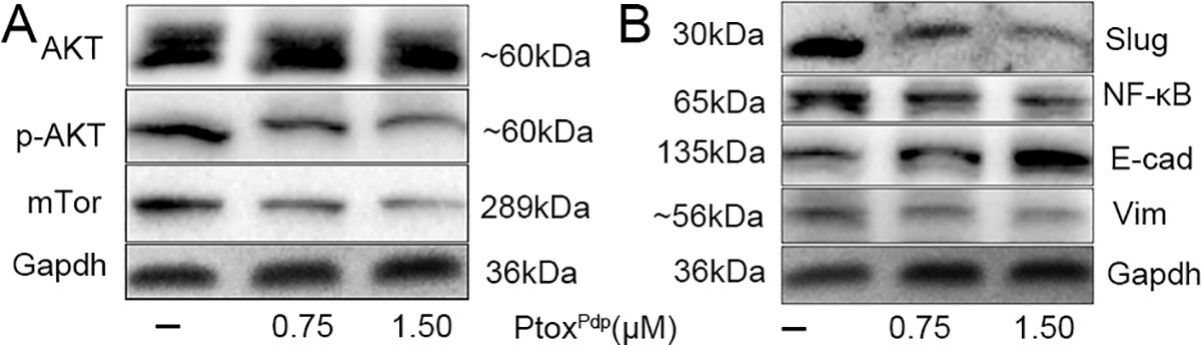
The regulation of the EMT and AKT signaling pathways by Ptox^Pdp^. (A) AKT/mTor regulation, the images of p-AKT, mTor and gapdh were from different parts of the same gel under different exposure time; (B) EMT regulation, the images of vimentin, E-cadherin and gapdh were from different parts of the same gel under different exposure time. The amount of proteins in different gels were loaded in each line was same. Vim = vimentin; E-cad = E-cadherin. The composite images were generated by using Adobe Photoshop.

The PI3K/AKT/mTOR pathway is an intracellular signaling pathway that regulates cell survival and proliferation [41]. Interestingly, tumor cells use the PI3K/Akt/mTOR signaling more than normal cells [42,43]. The fact that Ptox^Pdp^ exhibited an anti-proliferative effect both *in vitro* and *in vivo* prompted us to investigate its mechanism of action in greater detail. Thus, the expression levels of AKT, phospho-AKT (as a measure of AKT activation) and mTOR were determined by western blotting. As shown in **Fig 8A**, the phosphorylation levels of AKT were decreased by Ptox^Pdp^ treatment and accordingly the expression levels of its downstream target mTOR were also suppressed, indicating that the anti-proliferative of Ptox^Pdp^ was through inhibition of the PI3K/AKT/mTOR pathway. A previous study has shown that both mTORC1 and mTORC2 regulate EMT, motility, and metastasis in colorectal cancer [44]; therefore, the downregulation of mTOR induced by Ptox^Pdp^ may therefore affect EMT status. In order to explore this aspect, changes in the protein levels of markers of the epithelium (E-cadherin) and mesenchymal cells (vimentin) were assessed. As expected, an upregulation of E-cadherin and a downregulation of vimentin were observed following exposure of HepG2 cells to Ptox^Pdp^, implying that Ptox^Pdp^ could inhibit EMT transition (**Fig 8B**). Furthermore, the levels of NF-κB and slug were also decreased, indicating that NF-κB/slug pathway was also involved in the EMT transition, consistent with previous reports [45,46].

## Discussion

Topoisomerases are ubiquitous enzymes that control DNA supercoiling and entanglement, and play an important role in cell growth. Because cancer cells grow faster than normal cells, the strategy of topoisomerase inhibition has been widely used in cancer therapy, despite its lack of selectivity. Etoposide (ETO), as a Topo II inhibitor is well-known, however its moderate efficacy, the development of drug resistance, and its toxic effects have limited its wide use in the clinic [46]. Therefore, new topoisomerases inhibitors that have lower toxicity are still required. In view of Topo II inhibition by DMEP, a number of structural modifications at position 4 in DMEP have been conducted, including esterification and amination [5,8–10,12]). For esterification, the carboxyl group was linked to either aromatic rings or non-aromatic rings, and an improved anti-proliferative effect was achieved compared to that of DMEP or ETO at the cell level [5]. It should be noted that although many modified derivatives of DMEP have been prepared and structure-activity relationships (SAR) have been explored, there is still a lack of a clear SAR that can guide drug design; therefore, more effort is needed to improve the activity and selectivity of Topo II inhibitors.

Cancer cells reside in specific tissues, alongside concomitant stroma cells, the ECM and other components of the ECM (referred to as the microenvironment) and these can have a profound effect on cancer development and metastasis [47]. Among associated cells, cancer associated macrophages, as scavenger of senile or necrotic cells, can provide enriched iron, cytokines, and proteinases for use by other cells. Given the presence of enriched transition metals and proteinases in the tumor microenvironment, metal chelators have been shown to disturb metal ion homeostasis, as well as metalloproteinase activity, resulting in a delay in the cell cycle or inhibition of metastasis. In the present study, we propose a novel strategy of targeting Topo II in the host cell, and metalloenzymes in the tumor microenvironment, using intravenous administration. To this end, a metal chelator, a dithiocarbamate unit, was introduced as a structural modification of DMEP to achieve these dual functions (**Fig 1**). An in vitro inhibition experiment revealed that Ptox^Pdp^ was as an effective Topo II inhibitor as etoposide. Furthermore, a theoretical simulation further supported these in vitro data, since Ptox^Pdp^ was found to bind to the DNA-Topo complex in a similar manner and with similar affinity as etoposide (**Fig 2**). Gelatinzymography indicated that Ptox^Pdp^ could also inhibit the activities of MMPs (**Fig 4E**). Approximately 90% of all cancer-related deaths arise as a result of metastasis. In the present study, the data from the wound scratch and transwell invasion assays demonstrated that Ptox^Pdp^ would be expected to significantly inhibit metastasis. And MMPs are traditionally associated with matrix remodeling, and in particular with cancer invasion. The downregulation of MMP-2, and MMP-9 from western blotting hinted that its anti-metastatic effect of Ptox^Pdp^ may correlate with MMP inhibition. Previous studies have revealed that many metal chelators exhibit significant MMP inhibition [48,49], and dithiocarbamates, as NF-κB inhibitors, have been shown to regulate MMP expression in several different cell lines [50,51]. However, Ptox^Pdp^ as a dithiocarbamate derivative repressed MMP-2, and MMP-9 expression in the liver cancer cell line was first observed (**Fig 4E**), this indicated that the structural unit of podophyllotoxin introduced to dithiocarbamate did not alter action pattern in regulation of MMPs. Taken together, these data strongly suggest that our design concept was realized.

Next, we evaluated inhibition of cell proliferation and colony formation by Ptox^Pdp^ (**Fig 3**), revealing that Ptox^Pdp^ owned significant antitumor activity. This has also been observed for other podophyllotoxin derivatives [4–10]. Since Ptox^Pdp^ owned excellent antitumor activity against liver cancer *in vitro*, whether similar activity in vivo required to be determined. Interestingly Ptox^Pdp^ was able to suppress xenograft tumor of HepG2 cell and superior to etoposide (**Fig 5**). This was an exciting result, which may be partly due to better Topo II inhibitory activity than etoposide. The experimental data both from in vitro and in vivo supported that Ptox^Pdp^ was a potent candidate of antitumor drug. In view of excellent anti-proliferative effect of Ptox^Pdp^, the underlying mechanism required to be further explored. It is well-known that many chemotherapeutic agents inhibit cell proliferation by generating excess ROS [52]. We anticipated that Ptox^Pdp^ might act as a chemotherapeutic agent, such as etoposide [53]. To this end, the ROS level in HepG2 cell was examined following exposure of the cells to Ptox^Pdp^ for different periods of time. As expected, Ptox^Pdp^ indeed increased ROS generation (**Fig 6A**) that mediated DNA fragmentation and apoptosis [54,55], thus the DNA fragmentation was determined further using a comet tail assay [56]. As showed in **Fig 6**, Ptox^Pdp^ treatment led to both DNA fragmentation (**Fig 6B**), and apoptosis. Additional evidence from both flow cytometry and Western blotting analysis further supported the involvement of apoptosis in the mechanism of action of Ptox^Pdp^ [57] (**Fig 6D**). Increased ROS levels can lead to an autophagic response; therefore, we assessed the effect of Ptox^Pdp^ on LMP because the upregulated bax may be transferred to lysosomal membranes [40]. As expected, changes in LMP were observed, implying that lysosomal cell death might also be involved in the mechanism of action of Ptox^Pdp^. As previously mentioned, ROS production can induce autophagy and this can be easily measured in cells by monitoring an increase in the levels of LC3-II. In the present study, an increase in LC3-II levels suggested that the anti-proliferative effect of Ptox^Pdp^ may also involve autophagy (**Fig 7**) [41]. However, the question whether the autophagy was due to changes in LMP or due to a cytotoxic effect induced by Ptox^Pdp^ will need to be clarified in future studies.

To understand profoundly the mechanism of action of Ptox^Pdp^, the engagement of potent signal pathway was further investigated. It is known that the PI3K/Akt/mTOR pathway plays a critical role in the proliferation, apoptosis, and metastasis of tumor development [58,59], the Ptox^Pdp^ might be through similar pathway. As expected, our data indicated that Ptox^Pdp^ can significantly downregulate phosphorylated AKT and mTOR (**Fig 8**), suggesting that the induction of apoptosis caused by Ptox^Pdp^ was mediated through the PI3K/AKT/mTOR pathway [60], which was consistent with that reported previously for other podophyllotxin derivative [61]. The epithelial-mesenchymal transition (EMT) is a physiological process, aberrant EMT activation contributes to cancer progression and metastasis [62,63]. Recent report showed that etoposide treatment could reverse TGF-β induced EMT [64]. As an etoposide analog, Ptox^Pdp^ would be expected to behave in a similar fashion with respect to the EMT, thus the effect of Ptox^Pdp^ on EMT was also investigated, as expected Ptox^Pdp^ could upregulate E-cadherin and downregulate vimentin (Fig 8A), suggesting that Ptox^Pdp^ had similar ability in inhibition of EMT, which correlated with downregulation of NF-κB and snail1, in accordance with results reported previously [65–68]. However, The EMT inhibition induced by Ptox^Pdp^ requires to be explored systematically in deep via observation of the change in morphology, immunofluorescence analysis in the presence or absence of TGF-β1, and the signal pathway engaged in the absence or presence of the inhibitor in the future project. In addition, whether the growth inhibition of Ptox^Pdp^ in vivo was achieved through influencing phenotype of tumor associated macrophagy (TAM) requires to be further determined [69]. Taken together these data indicated that the Ptox^Pdp^ exhibited antitumor activity via multiple signaling pathways.

## Conclusions

In conclusion, Ptox^Pdp^ exhibited a dual functions both inhibiting Topo II and MMPs. It could inhibit growth of liver cancer cells both *in vitro* and *in vivo*. Mechanistically, the action of Ptox^Pdp^ involved apoptosis and PI3K/AKT/mTOR pathway. Furthermore, Ptox^Pdp^ also inhibited EMT, which may be achieved by downregulating the NF-κB/Snail. Taken together, our findings indicate that Ptox^Pdp^ is a promising antitumor drug for potent use in chemotherapy. However, extensive investigations, both *in vitro* and *in vivo*, are required in future studies.

The characterization of Ptox^Pdp^, its copper chelating ability and ROS generation were compiled in supporting information.

## Supporting information

S1 Fig Purity of Ptox^Pdp^ was determined by HPLC. (TIF)

S2 Fig ^1^HNMR spectrum of Ptox^Pdp^. (TIF)

S3 Fig ^13^CNMR spectrum of Ptox^Pdp^. (TIF)

S4 Fig Mass spectrum of Ptox^Pdp^. (TIF)

S5 Fig Molecular simulation of etoposide and Ptox^Pdp^. (a) Comparison of docked etoposide with crystalized etoposide in DNA-topoisomerase complex; (b) Comparison of docked etoposide with docked Ptox^Pdp^ in DNA-topoisomerase complex.
(TIF)

S6 Fig The podophyllotoxin and etoposide induced growth inhibition of HepG2 cells. (TIF)

S7 Fig The interaction of Ptox^Pdp^ with copper ion: (a) spectral changes of Ptox^Pdp^ when addition of CuCl_2_ in acetonitrile; (b) ratio of Ptox^Pdp^/Cu was determined based on spectral change; (c) the color change when Ptox^Pdp^ mixed with copper ion in aqueous solution.
(TIF)

S8 Fig Ptox^Pdp^ induced ROS production at different time period: (a) 6h; (b) 12h; (c) 24h. DMSO group (A1, B1 and C1), 1.56 μM Ptox^Pdp^ (A2,B2, and C2); 3.12 μM (A3, B3 and C3).
(TIF)

S1 File (DOCX)
