# Supplementary figures and images for: Antitumor activity of a novel dual functional podophyllotoxin derivative involved PI3K/AKT/mTOR pathway
Source: PLoS One. 2019 Sep 26;14(9):e0215886. doi: 10.1371/journal.pone.0215886 (PMC6763125; doi:10.1371/journal.pone.0215886)

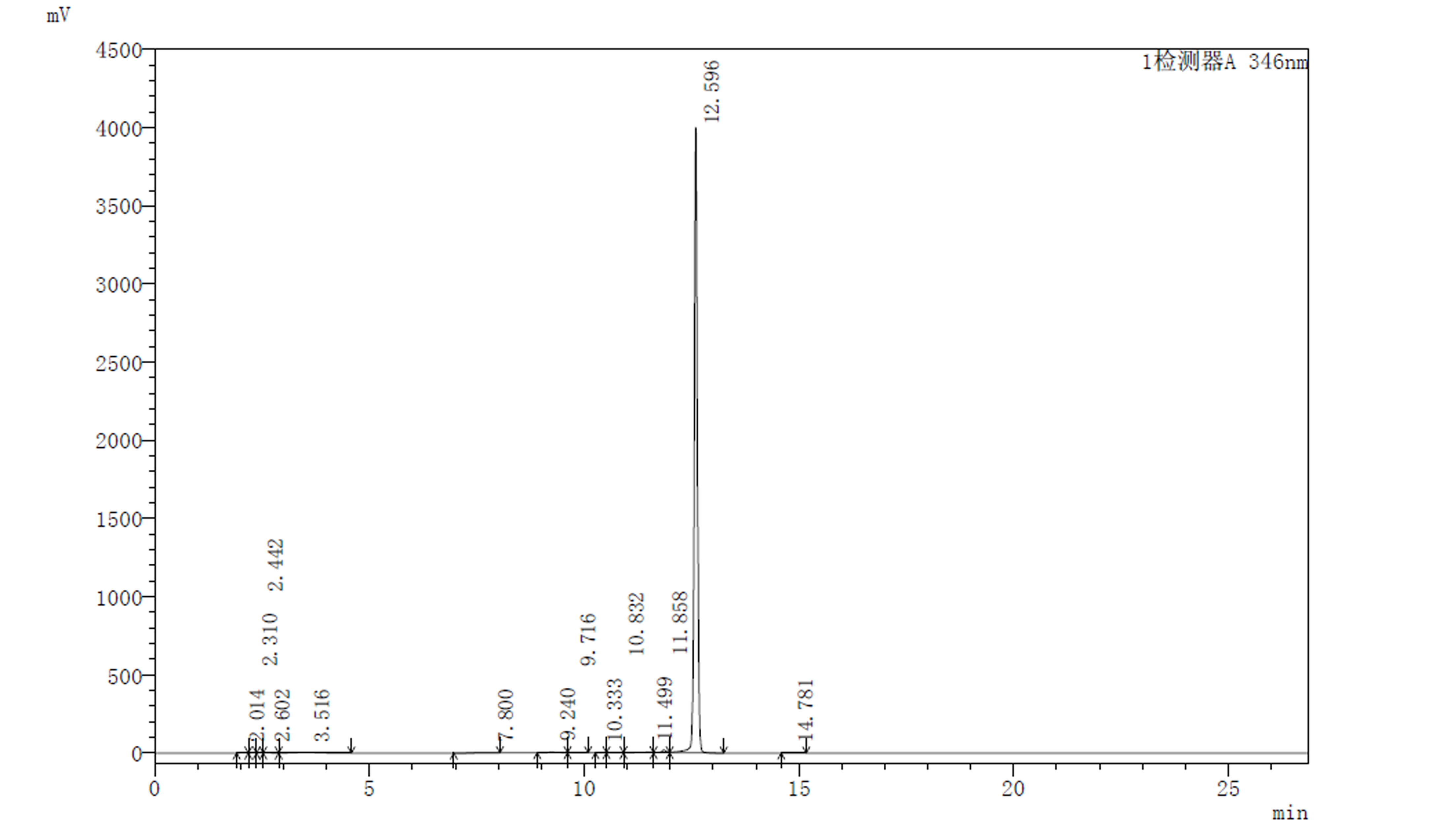

Supplement: S1 Fig — (TIF) [file pone.0215886.s001.tif]

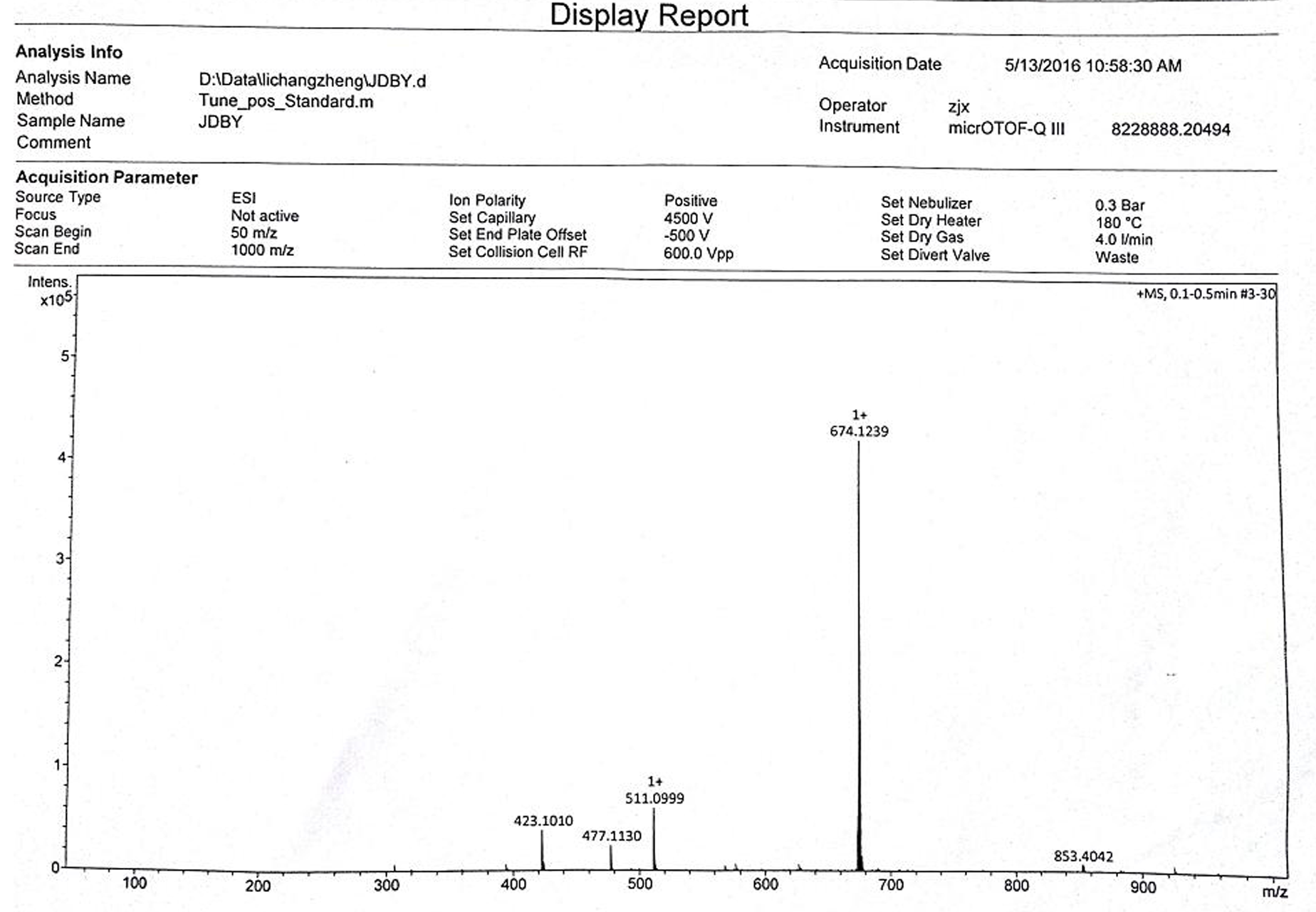

Supplement: S4 Fig — (TIF) [file pone.0215886.s004.tif]

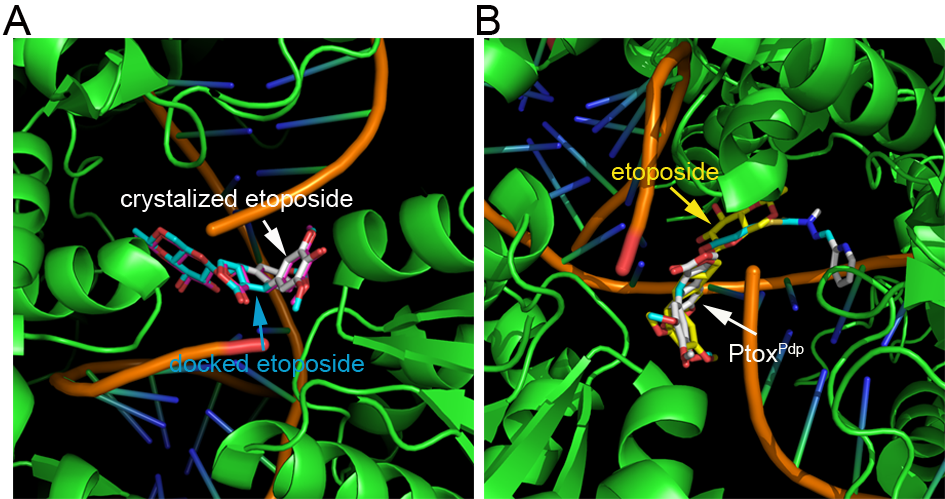

Supplement: S5 Fig — (a) Comparison of docked etoposide with crystalized etoposide in DNA-topoisomerase complex; (b) Comparison of docked etoposide with docked PtoxPdp in DNA-topoisomerase complex. (TIF) [file pone.0215886.s005.tif]

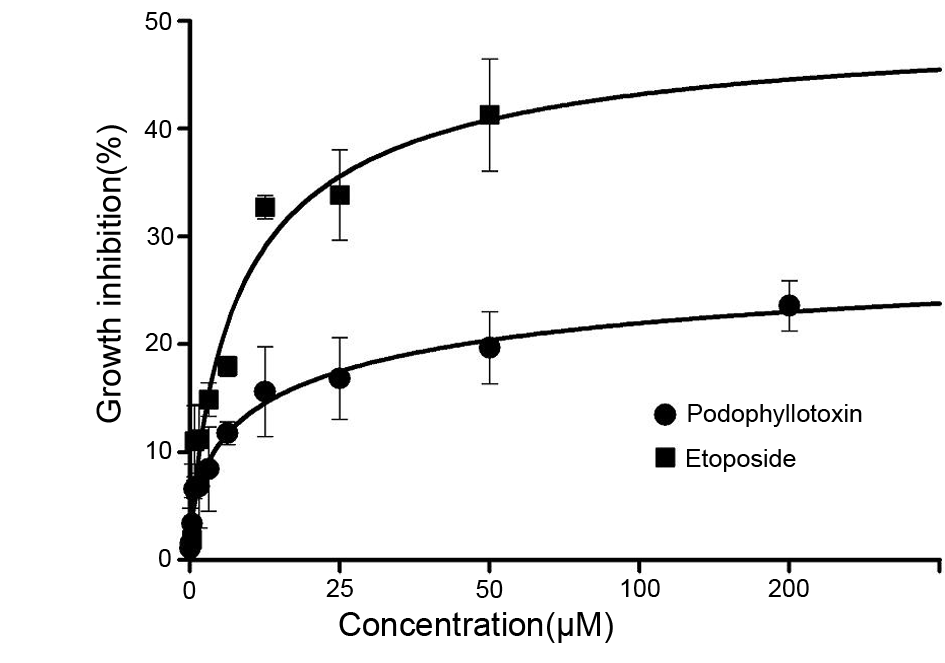

Supplement: S6 Fig — (TIF) [file pone.0215886.s006.tif]

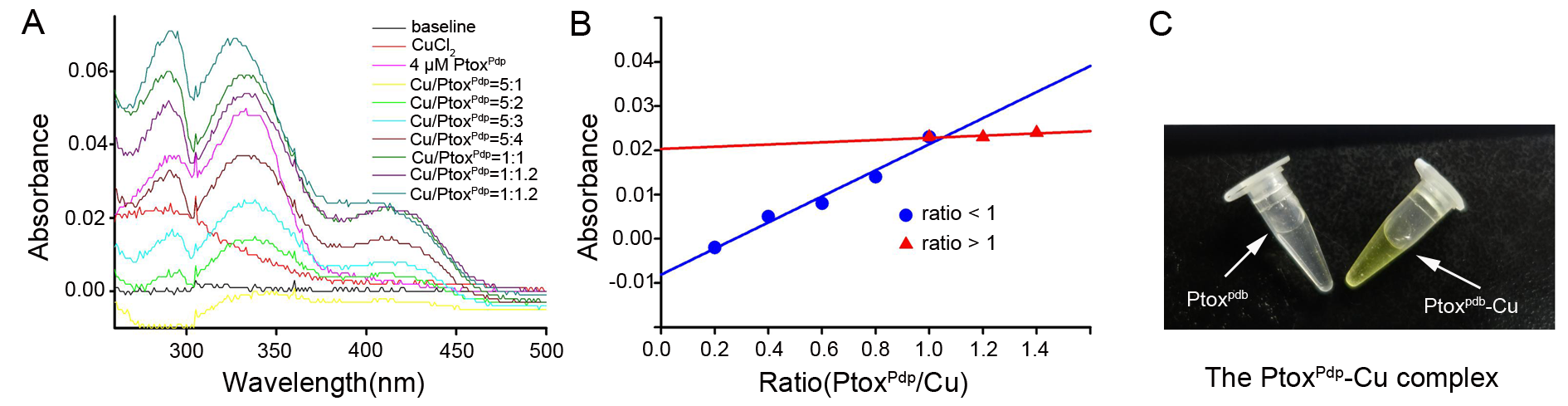

Supplement: S7 Fig — The interaction of PtoxPdp with copper ion: (a) spectral changes of PtoxPdp when addition of CuCl2 in acetonitrile; (b) ratio of PtoxPdp/Cu was determined based on spectral change; (c) the color change when PtoxPdp mixed with copper ion in aqueous solution. (TIF) [file pone.0215886.s007.tif]

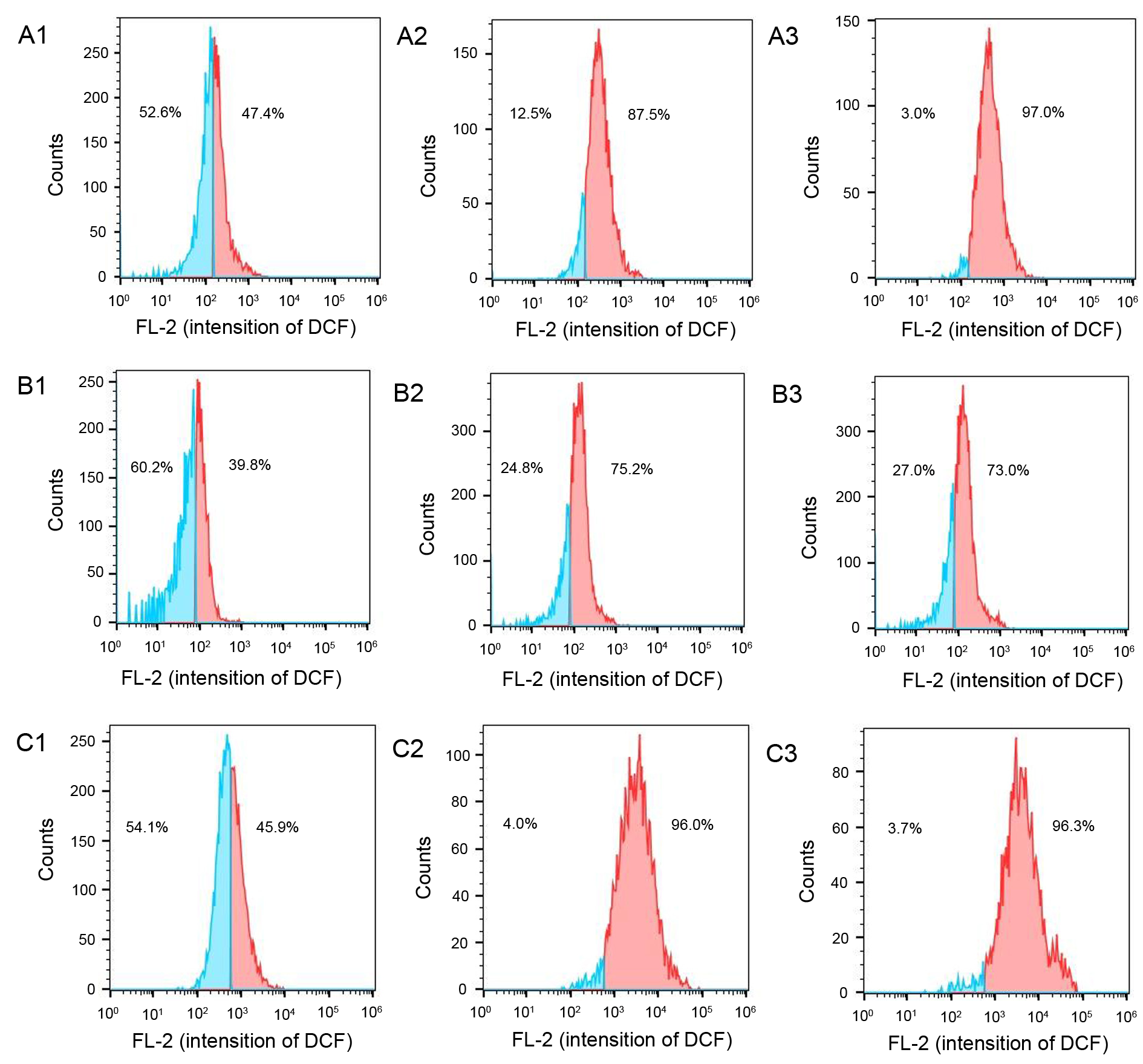

Supplement: S8 Fig — PtoxPdp induced ROS production at different time period: (a) 6h; (b) 12h; (c) 24h. DMSO group (A1, B1 and C1), 1.56 μM PtoxPdp (A2,B2, and C2); 3.12 μM (A3, B3 and C3). (TIF) [file pone.0215886.s008.tif]
