# Supplementary material for: Antitumor activity of a novel dual functional podophyllotoxin derivative involved PI3K/AKT/mTOR pathway
Source: PLoS One. 2019 Sep 26;14(9):e0215886. doi: 10.1371/journal.pone.0215886 (PMC6763125; doi:10.1371/journal.pone.0215886)
Supplement: S1 File — (DOCX) [file pone.0215886.s009.docx]

**Supplementary Material**

**Purity of Ptox^Pdp^ was determined by HPLC and NMR**

HPLC (Shimadzu Corpation, Kyoto, Japan) isolation(gradient: 20-50% solvent B within 10 min, following increased to 100% in 10 min, and decreased to 20% in 2 min. and keep same percent to 30 min). Solvent A: water plus 0.1% TFA; solvent B: acetonitrile plus 0.1% TFA)

**S1 Fig.** Purity of Ptox^Pdp^ was determined by HPLC.

**^1^HNMR spectrum of Ptox^Pdp^**

**S2 Fig.** ^1^HNMR spectrum of Ptox^Pdp^.

**^13^CNMR spectrum of Ptox^Pdp^.**

**S3 Fig.** ^13^CNMR spectrum of Ptox^Pdp^.

**MS of Ptox^Pdp^**

**S4 Fig.** Mass spectrum of Ptox^Pdp^.

**Conformation comparison of etoposide with Ptox^Pdp^ in catalytic center of DNA Topoisomerase II**

As shown in S5 Fig, the re-docked etoposide was fully superposed to crystalized etoposide, indicating that the protocol used was feasible (S5A Fig). Thus the conformation comparison of docked Ptox^Pdp^ with etoposide was further conducted, clearly the Ptox part both from etoposide and Ptox^Pdp^ were located in similar position (S5B Fig).

**S5 Fig.** Molecular simulation of etoposide and Ptox^Pdp^. (a) Comparison of docked etoposide with crystalized etoposide in DNA-topoisomerase complex; (b) Comparison of docked etoposide with docked Ptox^Pdp^ in DNA-topoisomerase complex.

**Ptox^Pdp^ exhibited enhanced cytotoxicity on HepG2 cell**

**S6 Fig.** The podophyllotoxin and etoposide induced growth inhibition of HepG2 cells.

**Ptox^Pdp^ can chelate copper ion**

The stoichiometry of the reaction between Ptox^Pdp^ and copper chloride was determined as previously described [1]. Briefly, 1mM Ptox^Pdp^ in 50 % DMSO and 1mM CuCl_2_ in water were prepared. Next, 0.04 ml of the CuCl_2_ solution was added to 5 ml volumetric flasks, then different volume of Ptox^Pdp^ (10, 20, 30, 40, 50, 60, 70, 80, 85, 90 µl) was also added to the each flask respectively, finally fixed volume at 5 ml by addition of 50 mM Tris-HCl buffer (pH 7.4). After mixing and equilibrium, the spectra were recorded on a Shimadzu-UV-2450 spectrophotometer (Shimadzu Co. Ltd, Suzhou, China).

**S7 Fig.** The interaction of Ptox^Pdp^ with copper ion: (a) spectral changes of Ptox^Pdp^ when addition of CuCl_2_ in acetonitrile; (b) ratio of Ptox^Pdp^/Cu was determined based on spectral change; (c) the color change when Ptox^Pdp^ mixed with copper ion in aqueous solution.

**Ptox^Pdp^ induced continuous ROS production**

**S8 Fig.** Ptox^Pdp^ induced ROS production at different time period: (a) 6h; (b) 12h; (c) 24h. DMSO group (A1, B1 and C1), 1.56 µM Ptox^Pdp^ (A2, B2 and C2); 3.12 µM (A3, B3 and C3).

1. Yang Y, Li C, Fu Y, Liu Y, Zhang Y, et al. Redox cycling of a copper complex with benzaldehyde nitrogen Mustard-2-pyridine carboxylic acid hydrazone contributes to its enhanced antitumor activity, but no change in the mechanism of action occurs after chelation. Oncol Rep 2016; 35: 1636–1644.
